# Supplementary material for: Are carbon emissions trading and green financial instruments synergistic? -Comprehensive quantitative research based on content analysis
Source: PLoS One. 2024 Mar 7;19(3):e0298601. doi: 10.1371/journal.pone.0298601 (PMC10919601; doi:10.1371/journal.pone.0298601)
Supplement: S1 Data — (ZIP) [file pone.0298601.s001.zip › Data/地级市Z_F工作报告.zip!/─■╧─/╬Γ╓╥╩╨/└┤╫╘╣1⁄2╓┌║┼í╛┬φ┐╦╩2╛▌═°í┐_2006.docx]

各位代表：

现在，我代表市人民政府向大会作工作报告，请各位代表与《吴忠市国民经济和社会发展“十一五”规划及2020年远景目标纲要(草案)》一同审议，并请市政协委员和其他列席人员提出意见。

一、“十五”简要回顾和2005年工作情况

第十个五年计划时期是我市经济社会发展进程中不平凡的五年。五年来，面对两次行政区划调整，区域经济结构、优势产业布局、资源分布状况发生重大变化的新市情，我们坚持奋斗目标再提升，发展速度再加快，工作激情再强化，众志诚城，攻坚克难，开拓创新，锐意奋进，统筹山川协调发展，全力推进工业化、城市化和农业产业化进程，顺利实现了“十五”计划预期的各项主要目标，全市呈现出经济繁荣、社会进步、民族团结、政治稳定的良好局面。经济实力持续提升。全市地区生产总值和地方财政收入年均增长11%以上，人均GDP迈上1000美元新台阶。经济结构渐趋合理，三次产业结构从“九五”末的24：48：28调整为16：52：32。乳品、羊绒、草畜、马铃薯、酿酒葡萄、设施蔬菜等特色产业不断发展壮大。各类农副产品加工企业发展到近2000家。粮食产量稳定在60万吨以上。五年累计完成人工造林520万亩，新增灌溉面积15万亩。依托“三山”荒地及资源优势，实施“园区上山、工业进园”。举全市之力规划和建设太阳山移民开发区“一号工程”，加快推动青铜峡新材料、牛首山镁合金等工业园区提升水平，形成了以能源电力、高载能新材料、机械仪表、农产品加工为主的优势特色工业产业体系。全市工业持续保持了总产值、增加值、销售收入、利润“四个同步增长”，总产值年均增长18%，对经济增长的贡献率比2000年提高7个百分点。第三产业蓬勃发展，增加值占GDP的比重比2000年提高2.4个百分点。城乡面貌明显改观。按照全市城市发展规划目标和建设滨河生态城市的要求，集中实施了一批重大项目工程。五年累计完成固定资产投资225亿元，年均增长30%。市区先后完成开元大道、“民居计划”、行政中心、盛元广场、滨河大道、迎宾大街等一批重点工程及集中供热、供水、排污等一批基础设施建设项目，使市区逐年变大、变新、变美。市区建成区面积由2000年的16平方公里扩大到23平方公里，城市人均新增公共绿地2.8平方米，城市化率提高11个百分点。各县(市、区)城镇面貌也发生了前所未有的变化，城镇集聚效应和辐射功能进一步增强。全市公路通车里程达4865公里，比2000年增加1114公里。改革开放纵深推进。改制国有企业45家，盘活国有资产8.9亿元，改制面达96%。农村税费、粮食流通、住房、水利产权等制度改革稳步推进，国库集中收付、收支两条线、会计集中核算、乡财县管和政府统一采购等财政管理改革取得成效。企业投资项目备案制、核准制正式实施。县乡机构改革基本完成，乡镇撤并率达40%。对外开放不断扩大，五年累计实际利用国外资金1.5亿美元，全市外贸进出口总额年均增长21%。人民生活不断改善。以养老、失业、医疗、工伤、生育为主的社会保障体系进一步健全，居民最低生活保障制度不断完善，基本养老金社会化发放率达100%，城镇登记失业率控制在4.5%以内。五年城镇累计新增就业7万余人，减少贫困人口10.6万人，输出劳务60万人，低保对象基本实现“应保尽保”。全市城镇居民可支配收入和农民人均纯收入年均分别增长8.5%和5%。社会事业全面进步。五年累计实施农业科技项目250多项，新技术改造项目26项，新产品开发项目17项，科技对经济增长的贡献率比2000年提高5个百分点。市区、青铜峡市通过“普九”复验，盐池、红寺堡通过“普九”验收，“两基”人口覆盖率比2000年提高15个百分点。高中阶段入学率、大专以上升学率、学前教育入园率和儿童入学率分别提高21.3、20.2、6.7和4.1个百分点。农村广播影视公共服务体系建设启动，8套免费电视节目农村覆盖率川区达80%以上、山区达40%以上。“非典”防治工作取得成效。文化体育活动蓬勃开展，医疗卫生事业健康发展。计划生育、土地管理、环境保护国策得到有效落实。精神文明和民主法制建设不断加强，依法行政、综合治理、反腐倡廉取得阶段性成效。统计、文史档案、外事侨务、残疾人、老龄、妇女儿童、民族宗教、人民武装、人民防空、防震减灾、气象预报等各项工作都迈上了新台阶。过去的五年，是我市经济社会持续快速发展、综合实力显著增强的五年，是城乡面貌发生显著变化、人民生活水平不断提高的五年，也是三个文明共同进步、各项社会事业全面发展的五年。“十五”时期取得的各项成就，谱写了我市发展史上的新篇章，为“十一五”发展奠定了坚实基础，增强了我们继续开拓前进的信心。

各位代表，刚刚过去的2005年，是“十五”时期的最后一年，也是确保“十五”计划顺利完成的关键一年。各级政府和全市人民在自治区党委、政府和市委的正确领导下，以邓小平理论和“三个代表”重要思想为指导，以开展保持共产党员先进性教育活动为动力，以建设宁夏经济次中心为目标，深入贯彻党的十六大和十六届四中、五中全会精神，全面落实科学发展观，团结奋进，开拓创新，圆满完成了市二届人大二次会议确定的各项目标任务，国民经济和社会事业呈现出良好的发展态势。预计全市完成地区生产总值108.3亿元，增长12.8%；全社会固定资产投资55亿元，增长9%；地方财政收入5.7亿元，增长11.2%；城镇居民人均可支配收入7412元，农民人均纯收入2825元，分别增长11.1%和4.7%。

——工业经济快速增长，运行质量明显提高。全市完成工业总产值143.5亿元、增加值43.2亿元，分别增长23.5%和22.6%。其中，地方工业完成产值75亿元、增加值22.8亿元、利润2.96亿元，分别增长23%、23%和22%，列入自治区考核的地方规模以上工业企业增加值、利润增速位居全区前列。重点支持的68个投资500万元以上的新建、技改、扩建项目有57个开工建设，40个建成投产，完成投资16.2亿元，新增产值18亿元。全市7个工业园区完成基础设施投资8967万元，申请入园项目109个。太阳山移民开发区开工建设各类项目29个，总投资2.4亿元，实际完成投资1.66亿元。成功对韦州小泉、红寺堡线驮石地区的五个区块煤炭资源探矿权进行了公开拍卖。深入开展“非公有制经济发展年”活动，非公有制工业企业完成产值66亿元、增加值20亿元、利润2.5亿元，分别增长30%、29.5%和22%。

——农业结构不断优化，农村经济稳步发展。全市农业实现总产值31.9亿元，增长1.2%。积极化解奶业市场风险，促进奶产业稳定增长。全市奶牛存栏11万头，新增2.6万头。盐池滩羊、滩鸡及同心、红寺堡栈羊、青铜峡生猪等特色产业规模不断扩大，全市肉牛、羊、生猪饲养量分别达到31万头、378万只和68万头。盐池县被命名为“中国滩羊之乡”。涝河桥牛羊肉批发市场被确定为“农业部定点市场”。粮食总产68.8万吨，增长3.6%。种植经济作物55万亩，优质饲草165万亩；设施瓜菜新增5000亩，酿酒葡萄新增1万亩。完成无公害农产品产地认定31宗，面积达20万亩。夏进乳业、御马酒业等企业龙头带动作用进一步增强。农村社会化服务及动物防疫体系不断健全，各类农村合作组织达107个，有效控制了高致病性禽流感等重大动物疫情发生。面对山区五十年不遇严重干旱，狠抓山区抗旱救灾、扶贫开发和劳务经济，39个整村推进项目稳步实施，75个重点扶贫村脱贫致富步伐加快。依托劳务品牌，输出劳务20.5万人，实现务工收入8.3亿元。

——消费需求稳中趋旺，第三产业彰显活力。全年实现社会消费品零售总额27.5亿元，增长12.4%，第三产业增加值占GDP的比重达30%。深入开展“重振商埠雄风大讨论”活动。启动实施涝河桥牛羊肉批发市场区域改造项目。成功举办吴忠市首届义乌小商品展销会、首届家具展销会、首届家电产品博览会、第二届房屋展览展示交易会等活动。义乌商贸城投入运营，新百家电连锁超市等商贸企业落户吴忠。房地产业稳步发展，商品房销售面积40万平方米，增长28.6%。承办、举办了宁夏旅游产品展示会和牛首山旅游文化节，全年接待游客64.5万人次，实现收入4012万元，分别增长84.3%和108.3%。

——滨河城市框架初显，城乡建设同步推进。全市安排城市建设项目219个，完成投资20.2亿元，其中市区开工建设项目96个，完成投资12.9亿元。编制完成市区西区、西花园片区控制性详细规划和滨河生态城市概念性规划。新建续建西一环等10条城市道路，总长8.7公里。开工建设市职业技术学院实训楼、中央大道商网等23个公建项目及盛世花园、塞上江南、明珠苑等13个住宅小区。吴忠宾馆成功平移，整合周边商业资源，启动实施商贸核心区改造建设工程。推进西泵站技改、城西集污等6个基础设施建设项目，完成投资1.4亿元。实施“一街四园七路”绿化工程，市区新增公共绿地22公顷。太阳山农民新居、孙家滩农民新村等8个“塞上农民新居”示范点新建农宅169栋。各县(市、区)开工建设城镇基础设施建设项目123项，完成投资7.3亿元。青铜峡市古峡东街与康乐小区、盐池县城市垃圾无害化处理与康泰花园、同心县利民街改造、红寺堡市政道路等重点项目建成投入使用。深入开展“物业管理年”活动，综合整治市区环境，城市形象和品位得到提升。

——招商引资取得实效，对外开放层次提升。全市签订各类招商引资项目334个，其中上海勤昌500万套汽车轮毂生产等145个项目开工建设，深圳安延2万吨甜玉米加工等83个项目建成投产，实际到位资金22.1亿元，增长33%。大力开展“招商引资年”活动，积极走出去与马来西亚、纳米比亚等国家建立友好合作关系。组团赴上海、义乌等地学习考察，参加上海投洽会、西安投洽会等经贸洽谈会，在义乌建立了政府办事处和劳务输出站。与上海联合产权交易所建立全面合作关系，由上交所帮助我市编制推进社会主义市场经济行动纲领项目并挂牌部分项目。成功承办陕甘川宁蒙毗邻地区经联会第二十届年会。完成政务大厅迁建整合。开通西部投资吴忠网。对外贸易扩大，全市外贸进出口总额达849万美元，增长23%。

——人民生活逐步改善，社会事业协调发展。全市城镇新增就业10419人，实现城镇下岗失业人员再就业1969人，其中“4050”人员700余人。城镇职工生育保险全面推行。城市居民最低生活保障提标扩面，农村特困户最低生活保障制度不断完善。人才劳动力市场和信息网络中心竣工投入使用。国有困难企业下岗职工基本生活保障向失业保险并轨试点工作成功实施，妥善解决土坡煤矿职工安置，全面盘活吴忠材机厂有效资产，基本完成市区9家企业改制任务，安置职工90%以上。积极推进科技特派员创业行动，各级各类科技园区达173个。率先在全区启动创建教育强区、强乡镇工作。全市15所回民中小学进入“百标”项目。顺利完成市区、青铜峡市部分学校布局调整，优质教育规模不断扩大。市紧急救援中心基本建成，传染病区投入使用。市区、盐池县新型农村合作医疗试点工作全面启动，农民参合率达90%以上。积极开展计划生育“三无”、“一无”乡镇创建活动，各县(市、区)均列入全区“少生快富”工程项目。环境污染防治与生态保护加强，土地、矿产、水资源得到有效保护和合理利用，促进了经济社会的可持续发展。

——精神文明建设加强，民主法制进程加快。认真贯彻《公民道德建设实施纲要》，文明社区、文明单位、文明家庭创建工作成效显著。成功举办市区元宵节花灯展、吴忠市首届合唱节、首届运动会，广泛开展“魅力吴忠”广场文化活动，凝聚了人心，振奋了精神。深入贯彻《行政许可法》和《全面推进依法行政实施纲要》，“四五”普法如期完成。市政府法律顾问团认真履行职责，为政府依法行政、依法决策提供了法律保障。在全市46个乡镇、街道设立法律援助工作站。基层民主政治建设不断加强，村民自治和社区居民自治有序推进。各级政府自觉依法接受人大及其常委会和人民政协的监督，市区两级人大代表议案12件、建议及意见104件和政协委员提案378件全部办复完毕。认真落实党风廉政建设责任制，深入开展纠风和执法检查专项治理，强化审计监督和行政监察，严肃查处违法违纪案件，促进了勤政廉政。国防动员教育和人民防空工作得到加强，“双拥”共建及“双拥”模范城创建工作成效明显，军政、军民关系进一步密切。党的民族宗教政策得到贯彻落实，平等、团结、互助的新型民族关系不断巩固和发展。坚持市级领导信访接待日制度，妥善解决群众来信来访。加强安全生产监督管理，深入开展专项整治，总体形势平稳。“平安吴忠”创建活动取得初步成效，青铜峡市、盐池县分别被自治区命名为首批“平安市”和首批“平安创建工作先进县”。强化社会治安综合治理，严厉打击各类违法犯罪活动，社会治安进一步好转，有力地维护了社会稳定和谐。

市区统一办公顺利实施，新的体制平稳运行。全市各级政府面对财政困难等诸多不利因素的影响，克服种种困难，积极创造条件，千方百计筹措资金，认真办理为民承诺的实事，有效解决了关系群众生产生活的一些困难和问题，收到了良好的社会效果。市区两级政府为民承诺的23件实事全面完成或落实，其中新建续建城市道路、建设垃圾中转站、解决城市中低收入家庭住房困难、综合整治黄河市区过境段、市区空气质量检测站、110、119、122“三台合一”报警系统、农村千户户用沼气工程、郭家桥乡五校合一综合教学大楼等17件全部完成；增设消防设施、新建和改造县乡村道路、农村劳务输出3件超额完成，分别为承诺任务的108%、118%和133%；开工建设明珠公园、迎客园、槐秀园、规划建设西部物流中心、迁建扩建第三中学3件全部落实。

回顾过去的工作，我们深切体会到，要实现跨越式发展，必须始终坚持加快发展不放松，牢固树立科学发展观，凝聚全市各方面力量，不断增强创新能力和争先进位意识，努力开创经济和社会发展的新局面；必须始终坚持解放思想，与时俱进，革除一切束缚发展的思想观念、体制弊端，营造良好的发展氛围，为加快发展提供强有力的环境支撑和制度保障；必须始终坚持以人为本、执政为民，时刻关注民生，把提高人民生活水平作为执政之本、为政之要，切实维护和发展广大人民群众的根本利益；必须始终坚持把稳定作为压倒一切的任务，正确处理改革、发展、稳定的关系，保护好、发挥好、引导好各方面发展的积极性，确保改革开放和现代化建设的顺利进行。这些基本做法和经验，在今后的工作中要继续坚持和发展。

各位代表，过去工作取得的成绩确实来之不易。这些成绩，靠得是党中央、自治区方针、政策的指引，凝结着全市人民的心血、智慧和力量，是方方面面关心支持、共同奋斗的结果。在此，我代表市人民政府，向全市广大工人、农民、知识分子、干部和驻吴部队、武警官兵、公安干警、中央及区属驻吴单位，向各位人大代表、政协委员，向各民主党派、各人民团体以及关心、支持吴忠发展的离退休老同志和社会各界人士，表示崇高的敬意和衷心的感谢！

我们也清醒地认识到,在前进的道路上还存在不少困难和问题。主要是：经济增长的质量和效益相对较低，固定资产投资增幅不高；工业结构性矛盾突出，产业链条短，企业自主创新能力弱，部分企业效益欠佳；农业特色产业规模不大，龙头企业与基地、农户联系不够紧密，抗旱和扶贫攻坚任务艰巨；城市建设历史欠帐较多，融资渠道不畅；招商引资的大项目不多，实际到位资金比例不够高，投资环境亟待改善；城乡居民收入持续增长的后劲乏力，就业再就业的压力仍未有效缓解；政府职能转变和依法行政工作仍需加强，政府工作效率、工作水平和创新服务的模式需要进一步提高和改善。我们将高度重视这些问题，在改革与发展的实践中认真加以解决。

二、“十一五”展望及2006年工作

从现在起到2010年，是我市加快建设宁夏经济次中心，为推进社会主义新农村、实现全面小康目标奠定基础的关键时期。根据《中共吴忠市委关于制定国民经济和社会发展第十一个五年规划的建议》精神，“十一五”时期我市经济社会发展的指导思想是：以邓小平理论和“三个代表”重要思想为指导,以全面建设小康社会为目标，以科学发展观统领经济社会发展全局，大力实施工业强市、商贸活市、新农村建设和科教兴市战略，全面推动社会主义物质文明、精神文明和政治文明共同进步，促进全市经济社会全面、协调、可持续发展。

奋斗目标是：地区生产总值达到210亿元，年均增长12%；财政收入达到9亿元，年均增长12.5%；固定资产投资累计达到500亿元，年均增长21%；城镇居民人均可支配收入突破1万元，年均增长7%，农民人均纯收入突破4000元，年均增长5%。人口自然增长率控制在12‰以内；城镇登记失业率控制在4%以内；城市化率达到47%左右。到2010年，市区打造成具有西部百强县实力的经济强区；青铜峡夯实争创全国百强县基础；盐池建成宁夏山区工业第一县；同心成为干旱带扶贫开发示范县；红寺堡三项主要经济指标(GDP、财政收入、农民人均纯收入)一年赶超一个山区县；全市上下共同努力建设宁夏经济次中心。

各位代表，今后五年规划是在认真分析国际国内形势和紧密联系市情的基础上拟定的，目标宏伟，催人奋进；任务艰巨，时不我待。我们一定要振奋精神，排除万难，开拓进取，为把我市建设成为民族团结、社会稳定、人民富裕、山川共同发展、区域特色鲜明、综合实力较强、充满现代文明气息的宁夏经济次中心打下坚实基础。

2006年是实施“十一五”规划的开局之年，扎扎实实地做好今年的工作,对于巩固“十五”发展成果，顺利实现“十一五”任务目标、加快推进全面建设小康社会意义十分重大。按照市委二届十一次全委(扩大)会议确定的“强工、重农、兴城、活商、聚势”，建设“经济发展又快又好、水韵吴忠和谐文明”的新吴忠的思路，今年我市国民经济和社会发展的主要预期目标是：地区生产总值达到120亿元，增长12%，其中市区45.8亿元，增长11%；全社会固定资产投资63亿元，增长15%，其中市区18亿元，增长8%；地方财政收入5.1亿元，可比增长12.5%，其中市区2.1亿元，可比增长11%；城镇居民人均可支配收入7900元，增长7%，其中市区8005元，增长8%；农民人均纯收入2950元，增长5%，其中市区4100元，增长5%；人口自然增长率控制在13‰以内；城镇登记失业率控制在4.5%以内。实现上述目标，着重抓好以下七个方面的工作：

（一）转变工业增长方式，提高工业经济运行质量

强化工业在全市经济发展中的主导地位，按照“园区主导，项目跟进，扶优扶强”的要求，加快“工业强市”进程，切实转变增长方式，提高增长质量和效益，力争工业总产值、规模以上企业增加值和利税都保持20%以上的增速。一是培育和发展优势骨干企业。围绕区属重点企业和能源电力项目，配合抓好青铝集团自备电厂、红寺堡风力发电等项目建设；围绕高载能新材料和机械制造项目，抓好金昱元技改、万兴车辆改装等项目建设；围绕生物发酵产业，抓好御马酒业、金樽酒业等企业扩建项目；围绕农副产品加工业，抓好夏进、中桦雪等10个产值过亿元的企业。力争年内培育销售收入过50亿元的企业1家、过10亿元的4家、过亿元的20家，全市工业总产值完成170亿元。二是提升工业园区聚合效益。在加快太阳山移民开发区基础设施建设的同时，以永安、韦一井田煤炭为重点，抓好矿产资源勘探开发，促进资源招商，力争年内引进投资亿元以上的企业2家，1000万元以上的企业6家。扩大市区金积、牛首山镁合金工业园区规模，提升青铜峡新材料、盐池东顺、同心羊绒、红寺堡工业园区招商引资的聚合力。力争年内全市工业园区开工建设亿元以上的项目5个，实现产值120亿元以上。三是大力发展非公有制经济。全面落实加快非公有制经济发展的各项政策措施，建立健全诚信评价、信用担保、人才培训、法律服务体系及各类行业协会组织，解决企业融资难、维权难和人才缺乏等问题。引导非公有制企业完善法人治理结构，通过参股、兼并、联合等方式，扶持一批成长性好的科技型非公有制企业，促使非公有制经济整体提升和群体拓展。力争全市中小工业企业达到1700家，实现地方工业产值90亿元。四是推进企业技术创新。以企业为依托，通过有偿服务、技术转让、引进及采用新技术、新设备、新工艺，加快技术创新、新产品开发及技术改造，提高企业的技术装备水平和产品科技含量，增强市场竞争力。加大对优势产业、龙头企业和拳头产品的扶持。重点抓好米来生物等65个投资500万元以上的技改扩建、新建和续建项目。实施品牌战略，鼓励企业培育自主品牌，并采取措施帮助企业保护品牌，提升工业发展水平。五是积极发展循环经济。以建设资源节约型、环境友好型社会为目标，围绕冶金、化工等高耗能行业，突出抓好节煤、节电、节油、节水和降耗，做好重点行业、领域、产业园区循环经济试点工作，形成资源开发、加工与废弃物回收利用的良性循环，力争工业万元增加值能耗低于3.37吨标准煤、水耗低于1380吨，工业水重复利用率达43%，固体废物利用率达37%。六是深化企业改革。积极做好印刷厂、造纸厂等企业改革后续工作，全力抓好益木公司等10家企业改革，继续争取国有困难企业职工基本生活保障向失业保险并轨试点的政策支持，妥善处理遗留问题，帮助企业重新焕发生机。

（二）高度重视和加强“三农”工作，扎实推进社会主义新农村建设

紧紧围绕“生产发展，生活宽裕，乡风文明，村容整洁，管理民主”的社会主义新农村建设目标要求，坚持因地制宜，分类指导，统筹山川，全面发展，稳定、完善和强化各项支农惠农政策，加快现代农业建设，壮大农村经济实力，切实增加农民收入。一是全力推进农业产业化经营。积极引导龙头企业与农户建立诚信为本、互惠互利的利益联结机制，走基地化、标准化、产业化的发展路子，提高农业生产的组织化程度。奶产业紧紧依托夏进公司等乳品加工企业，加快以“西部乳都”优质奶牛核心区为主的奶牛园区建设，强力推进“奶牛出户入园上挤奶台”工程，力争奶牛存栏达到13万头以上，入园率达到60%以上；牛羊肉产业依托宇华肉联和涝河桥牛羊肉市场，扩大品牌优势，力争肉牛饲养量达到35万头、羊400万只。稳步扩大生猪、家禽等特色养殖规模，力争生猪饲养量达到90万头、家禽700万只。全市畜牧业产值实现16亿元；优质粮食产业依托万胜生物、米来生物、嘉禾粮油等龙头企业，优化品种，扩大规模，力争优质水稻种植面积达到18万亩，优质小麦、优质专用玉米和优质马铃薯各40万亩；依托深圳东部集团、御马酒业和科冕公司，加快以孙家滩地区为核心的高酸苹果、青铜峡和红寺堡为主的葡萄基地建设，力争高酸苹果达到3万亩、酿酒葡萄5万亩。大力发展特色农业，重点加快吴忠国家农业科技园区种植业核心区和市区现代农业示范县建设，辐射带动全市无公害果蔬发展到15万亩，优质中药材5万亩。推进林草工程建设，巩固退耕还林、封山禁牧成果，种植优质牧草200万亩。二是加强农业基础设施建设。坚持不懈地抓好农田水利基本建设，实行沟、渠、田、林、路综合治理。重点实施盐、同、红境内小流域综合治理、草原保护和围栏建设等项目，提高中部干旱带抗旱能力。加快实施汉延渠与惠农渠合并、唐徕渠与西干渠合并、西夏渠开挖工程。三是大力发展劳务经济。按照培养一代新农民的要求，全面实施“阳光工程”、“百万农民培训工程”，建立健全劳务培训、信息中介和输出工作的长效机制。大力培养技术人才，扩大品牌优势，力争年内培训农民25万人，输出劳务18万人，实现劳务收入8亿元以上。四是抓好新农村规划和试点示范。科学制定新农村建设总体规划，积极开展试点示范。继续推进“塞上农民新居”工程建设，重点抓好太阳山农民新居二期、东塔寺乡柴园农民新村等6个示范点。加快农村道路、能源、通讯、安全饮水、庄点整治等公用事业建设，切实改善农村生产条件和人居环境。年内全市新建、改建农村等级公路260公里。五是加快山区脱贫致富步伐。实施扬黄扶贫扩灌项目，加快下马关、孙家滩引水工程建设，新开发耕地10万亩。积极推广秋春覆膜种植技术，大力发展抗旱避灾和节水农业，积极向节水型社会目标迈进。抓住中盐高速、中太银铁路开工建设的机遇，加快推进开发式扶贫。认真实施中部干旱带人畜饮水项目，加快推进“千村扶贫”、“整村推进”、山区危房危窑改造工程，继续开展市直机关定点帮扶工作。

（三）加快城市化进程，全力打造滨河生态城市

按照“依托黄河，相向发展，道路连城，生态扩城”的要求，加快滨河生态城市建设，提升县城和中心集镇的发展水平。全市安排重点建设项目92项，总投资20亿元，建筑面积134万平方米，力争城市化率达到38%，其中市区安排40项，总投资10亿元，建筑面积82万平方米，城市化率达到50%。一是加快重点项目建设。重点抓好吴忠公路黄河大桥项目的立项和宁东基地古窑子经市区至青铜峡“东西大通道”工程的前期论证、立项工作，争取早日开工建设。积极做好滨河大道主干线线型调整、滨河大道三期、黄河两岸连接线等工程项目。新建续建西一环南段、明珠路西段打通等6条城市道路，启动建设金积东大桥至滨河大道道路，为拉大城市框架、建设滨河生态城市奠定基础。抓好迎宾大街向南打通、明珠广场改造、城市集污续建、胜利西街集中供热、供水供暖分户改造等基础设施建设项目。新建续建吴忠汽车站、地税大厦等16个公建项目和华港小区、星河锦城二期等10个住宅小区。实施明珠公园、秦渠公园二期、开元大道A段南侧、西一环等园林绿化工程建设项目。二是推进县城及中心集镇建设。继续改造提升主要街区，完善基础设施，增强服务功能，提升城市聚集效能，切实抓好青铜峡市中庄至大坝公路、汉坝街与古峡街两条连接道路，同心县西北环路西段改造，盐池县革命历史纪念馆、盐州园，红寺堡开发区综合市场改造、政府街东段等项目建设。加快金积、峡口、惠安堡、韦州、太阳山等一批中心集镇建设，培育一批独具风格、集聚产业的新型小城镇。三是增强城市发展活力。发挥市场配置资源的基础性作用，激活土地、资本等要素，加快推进城市公用事业改革。建立多元化的投融资机制，鼓励引导企业、个人采取自建、公办民营等方式建设和经营供排水、集中供热等公用基础设施，切实增强城镇综合服务功能。挖掘城市潜能，完善城市经济带动、产业集聚、科教传播、文化服务功能，进一步提升城市品位和发展水平。

（四）加快发展现代商贸流通业，培植新的经济增长点

坚定不移地把发展现代商贸流通业作为促进经济增长的主要突破口，大力开展“现代商贸流通经济发展年”活动，完善市场功能，整合商贸资源，发挥商贸优势，打造商贸名城，力争全社会消费品零售总额达到30亿元，增长11%以上。一是壮大商贸物流业。整合提升专业批发市场功能，精心培育盐池商业广场、红寺堡罗山商城、同心羊绒和牛羊肉交易市场等特色专业市场。改造完善市区以涝河桥市场为依托的牛羊肉片区、以东郊市场为依托的蔬菜批发片区等“五大物流片区”，争取启动建设西部物流中心项目，打造新的商贸流通业发展平台。加快推进商贸核心区改造，规划建设吴忠国际商贸大厦。借鉴义乌经营理念，扩大双方交流合作，鼓励发展小商品批发业，力争尽快形成规模。坚持划行归市，发展各类商贸专业街和品牌名店专营街。二是激活特色旅游业。坚持“东中西三线”推进，东线开发陕甘宁边区盐池县和红军西征途经地同心县的红色经典游，中线开发古城湾、市区街心食府、古灵州遗址等民俗风情和历史文化游，西线开发提升青铜峡金沙湾、鸟岛、一百零八塔、罗山自然生态保护区、牛首山寺庙群等黄河沿线自然风光游，着力培育打造精品旅游线路。全年旅游收入增长25%以上。

（五）加大招商引资力度，全方位推进对外开放

坚持走出去与请进来相结合，内资外资一起引，通过“上争、内聚、外引”，力促招商引资工作再上新水平。全年招商引资实际到位资金22亿元以上，引进亿元以上项目10个，5000万元以上项目15个。一是坚定不移地实施开放带动战略。抓住国家对欠发达地区、革命老区和少数民族地区实施政策倾斜的机遇，千方百计向上争取国家、自治区国债项目及专项资金支持，加快城市排污、垃圾处理、乡村公路等基础设施建设。积极主动承接东部地区产业梯度转移，争取沿海地区企业、资金来我市创办农副产品加工业和绿色环保型企业，建立零部件或初级产品生产基地。密切与东南沿海地区、友好城市、经济协作组织的横向联系，充分利用当地企业的资金、市场和信息等优势，合作构建我市农副产品、特色食品等产品营销网络。加强与上海联合产权交易所交流合作，认真对接落实合作项目，不断扩大合作领域，提高合作水平。引导和鼓励本市企业以商招商、以企引企，内引外联，优势互补。二是强化招商引资实效。建立健全严格的考核机制和奖惩措施，进一步明确招商引资责任，着力提高项目的签约率、开工率和资金到位率，确保项目引得进、留得住。按照“瞄准项目抓洽谈，洽谈项目抓签约，签约项目抓开工，开工项目抓进度，建成项目抓效益”的要求，充分利用我市企业存量资产多、劳动力充裕、资源丰富等优势，打好资源牌、商贸牌、绿色牌、滨河城市牌、产权交易牌，吸引外资参与我市企业改造、农副产品加工、城镇基础设施建设及社会公用事业改革等领域。重点抓好天净公司投资4.3亿元的5万千瓦风力发电、宇华公司投资1.5亿元的“沙漠王子”肉食品配送中心等15个项目的落实工作。三是整治优化投资环境。加快行政审批改革，清理行政许可事项，强化政务大厅集中审批，实行行政审批一门受理，全程代理，阳光操作。认真落实招商引资的各项政策，积极搞好协调服务，坚决兑现对客商的承诺，严肃查处欺坑外地客商的不法行为，切实维护外来投资者的合法权益，树立我市对外开放的新形象。

（六）坚持以人为本，努力构建社会主义和谐社会

在加快经济发展的同时，时刻关注民生，高度重视发展社会事业，加强精神文明和民主法制建设，推进经济、社会和人的全面发展，加快向和谐社会的目标迈进。

加强就业再就业和社会保障工作。实施积极的就业再就业政策，把发展服务业、中小企业、劳动密集型企业、非公有制经济、劳务输出等作为扩大就业的主要方向，增加就业岗位，开发公益性岗位，强化政府对就业的指导和服务。加强职业培训特别是失地农民培训，提高劳动者就业技能和创新能力，年内实现城镇新增就业1万人，其中下岗失业人员再就业1000人。积极做好“两个确保”和“三条保障线”的衔接。全年养老、医疗、失业、工伤、生育保险人数达到20万人。加强劳动保障监察执法工作，切实保护劳动者合法权益。完善自然灾害救助、农村社会救助、城市社会救助制度，建立健全扶贫解困的长效工作机制。

大力发展科技教育事业。完善鼓励科技创新的财税、金融、用地和人才等政策，大力开展技术合作与交流，建立以企业为主导、产学研相结合的创新机制，提高区域自主创新能力和成果转化能力。围绕主导产业和优势特色产业，实施一批科技项目，组织开展科技攻关、技术开发。深入实施科技特派员创业行动，推进科技体制创新。认真做好科技部对国家农业科技园区的达标验收工作。按照“巩固基础，优化结构，城乡协调，优质均衡”的思路，加快推进川区教育强县、强乡（镇）和基本普及高中阶段教育进程，完成同心县“两基”攻坚任务，巩固提高“普九”成果。积极整合教育资源，继续实施学校布局结构调整，促进学前教育、基础教育、特殊教育和成人教育事业的均衡、协调发展。发挥吴忠职业技术学院、青铜峡职教中心作用，大力发展职业技术教育。认真落实“两免一补”政策，高度关注弱势群体教育，切实解决好进城务工家庭、农村流动人口和低保家庭子女入学问题。

推进文化、体育、卫生等事业发展。大力开展社区文化、广场文化、乡村文化、校园文化等群众性文化活动，巩固提升宣传文化体育中心户创建工作。实施文艺精品工程，挖掘和弘扬吴忠优秀历史文化，扶持具有地方特色、民族特色和时代精神的文化产品。广泛开展全民健身运动，努力提高竞技体育水平。积极做好市区、青铜峡市、盐池县农村新型合作医疗制度试点工作，争取同心县和红寺堡开发区列入试点县(区)，高度重视并逐步解决群众看病难、看病贵的问题。加强医疗市场监管，调整医疗机构布局，规范县、乡、村三级医疗预防保健网络，完善疾病控制、医疗救治、卫生监督体系，年内完成20个乡镇卫生院和一批村卫生室改扩建任务。维护妇女、儿童合法权益，关心支持老龄、残疾人事业。继续重视做好统计、文史档案、外事侨务、广播电视、防震减灾、气象预报等工作。

促进人与资源、环境的协调发展。加强人口与计划生育工作，推进“少生快富”工程和奖励扶助制度，深入开展“三无”和“一无”乡镇创建活动，年内实现三分之一乡镇达标。深化环保专项治理，加大污染防治力度，加强生态环境保护，严肃查处各种违法行为，切实改善城乡环境质量。依法保护和合理开发水、土地、矿产、森林、草原等自然资源，建立健全资源有偿使用制度，严厉打击滥采乱挖等非法行为，提高资源永续利用水平，推进经济社会可持续发展。

稳步推进各项改革。加快行政管理体制改革，全面完成县乡机构改革和人员分流，稳步推进事业单位改革。积极推行市政水务改革试点，加快供销合作社改革。深化农村税费配套改革，推进灌区水费改革进程。加快财税管理体制改革，完善地方财税收入征管办法，优化财税环境，强化稽查监督，加强收入征管，深化和推进部门预算、收支两条线、国库集中支付、政府采购、乡财县管等各项财政配套改革。

加强精神文明和民主法制建设。深入实施公民道德建设工程，推进文明城镇、文明社区、文明乡村和文明户创建活动，开展文化扶贫和“三下乡”、“四进社区”、“志愿者行动”等活动，提高全社会文明程度。拓展社区服务，改善社区环境，推进社区自治，建设和谐社区。加快依法治市进程，启动“五五”普法工作。推进基层司法所规范化建设，加强律师、公证和法律援助等工作。认真执行人民代表大会及其常委会的决定决议，积极支持人民政协发挥政治协商、民主监督和参政议政作用，认真办理人民代表议案、建议、意见和政协委员提案。密切与各民主党派、人民群众团体和社会各界的联系，不断拓宽社情民意的反映渠道。扩大基层民主，推进政务、村务、厂务、校务公开。认真落实党的民族宗教政策，依法加强民族宗教事务管理，切实做好新时期民族宗教工作。加强国防动员教育、人民防空和民兵预备役建设，巩固“双拥”成果，增进军政、军民团结。健全矛盾纠纷排查调处机制，强化人民调解和信访工作，积极预防并妥善处理人民内部矛盾和群体性事件。高度重视安全生产，加强食品药品安全，严格落实安全生产责任制，防止大的事故发生，保障人民生命财产安全。深化“平安吴忠”创建活动，认真落实社会治安综合治理的各项措施与维护稳定责任制，依法严厉打击各类违法犯罪，深入开展“人民禁毒战争”和“无毒社区、无毒学校、无毒单位、无毒村庄”创建活动，坚决扫除社会丑恶现象，创造更加稳定和谐的社会环境。

（七）加强政府自身建设，不断提高行政能力

完成今年的各项任务，实现“十一五”发展宏伟目标，必须按照“行为规范，运转协调，公正透明，廉洁高效”的要求，切实加强政府自身建设，努力把政府工作提高到一个新的水平。

转变职能，创建高效运转的管理体制。确立“管理就是服务”、“服务就是竞争力”的理念，强化政府公共事务管理职能，发挥市场配置资源的基础性作用，切实解决越位、缺位和错位的问题。建立健全各种突发事件应急机制，提高政府应对公共危机的能力。健全决策方式、规范决策程序、强化决策责任，提高决策的透明度和公众参与度，做到科学决策、民主决策。简化办事程序，提高机关效能，分工负责，团结协作，推动政府工作的高效便捷和协调运转。

依法行政，营造依法治市的浓厚氛围。深入贯彻《行政许可法》，全面清理行政许可规定和实施主体，严格执行规范性文件前置审查和备案制度。加快建立权责明确、行为规范、监督有效、保障有力的执法体制，完善行政执法责任制、评议考核制和过错责任追究制，增强政府工作人员依法行政的观念，做到规范执法、文明执法。加强行政执法监督检查和行政复议工作，认真落实行政赔偿制度，切实维护行政相对人的合法权益。

勤政为民，发扬求真务实的工作作风。牢固树立科学的发展观和正确的政绩观，坚持一切从实际出发，按客观规律办事，既要积极进取，又要量力而行，不盲目攀比。坚持重实际、说实话、办实事、求实效，不图虚名，不务虚功，确保好事办实，实事办好。大力推进政府信用建设，有诺必践，有错必纠，确保政府承诺落到实处。进一步改进工作作风，密切与群众的联系，精简会议，压缩文件，减少应酬，用更多的时间和精力，深入基层，深入群众，调查研究。继续做好为民承诺办理的实事，切实解决群众关注的热点、难点问题。加强责任落实，强化政务督查，严格兑现奖惩，进一步健全抓落实的工作机制。

从严治政，树立清正廉洁的公仆形象。认真学习贯彻《公务员法》，忠于职守，勤奋工作，清正廉洁，公道正派，努力造就一支政治坚定、业务精通、作风优良、廉洁奉公的高素质公务员队伍。大力倡导艰苦奋斗、勤俭节约的光荣传统，坚持勤俭办一切事业。认真落实中纪委六次全会精神和廉洁从政的各项规定，建立健全教育、制度、监督并重的预防和惩治腐败体系，加强行政监察、审计监督，推行“阳光行政”，坚决纠正部门和行业不正之风，努力促进政风行风进一步好转。

各位代表，全市改革开放和现代化建设正处于关键时期，我们深知任务艰巨，责任重大。让我们高举邓小平理论和“三个代表”重要思想伟大旗帜，紧密团结在以胡锦涛同志为总书记的党中央周围，在自治区党委、政府和市委的坚强领导下，坚定信心，振奋精神，迎难而上，扎实工作，为建设宁夏经济次中心，开创“十一五”时期经济社会发展的新局面而努力奋斗！
